# Identification and validation of a classifier based on hub aging-related genes and aging subtypes correlation with immune microenvironment for periodontitis

**DOI:** 10.3389/fimmu.2022.1042484

**Published:** 2022-11-01

**Authors:** Limin Peng, Hang Chen, Zhenxiang Wang, Yujuan He, Xiaonan Zhang

**Affiliations:** ^1^ College of Stomatology, Chongqing Medical University, Chongqing, China; ^2^ Chongqing Municipal Key Laboratory of Oral Biomedical Engineering of Higher Education, Chongqing, China; ^3^ Chongqing Key Laboratory of Oral Diseases and Biomedical Sciences, Chongqing, China; ^4^ Department of Laboratory Medicine, Key Laboratory of Diagnostic Medicine (Ministry of Education), Chongqing Medical University, Chongqing, China

**Keywords:** aging-related genes, diagnosis, immune microenvironment, periodontitis, bioinformatics

## Abstract

**Background:**

Periodontitis (PD), an age-related disease, is characterized by inflammatory periodontal tissue loss, and with the general aging of the global population, the burden of PD is becoming a major health concern. Nevertheless, the mechanism underlying this phenomenon remains indistinct. We aimed to develop a classification model for PD and explore the relationship between aging subtypes and the immune microenvironment for PD based on bioinformatics analysis.

**Materials and Methods:**

The PD-related datasets were acquired from the Gene Expression Omnibus (GEO) database, and aging-related genes (ARGs) were obtained from the Human Aging Genomic Resources (HAGR). Four machine learning algorithms were applied to screen out the hub ARGs. Then, an artificial neural network (ANN) model was constructed and the accuracy of the model was validated by receiver operating characteristic (ROC) curve analysis. The clinical effect of the model was evaluated by decision curve analysis (DCA). Consensus clustering was employed to determine the aging expression subtypes. A series of bioinformatics analyses were performed to explore the PD immune microenvironment and its subtypes. The hub aging-related modules were defined using weighted correlation network analysis (WGCNA).

**Results:**

Twenty-seven differentially expressed ARGs were dysregulated and a classifier based on four hub ARGs (BLM, FOS, IGFBP3, and PDGFRB) was constructed to diagnose PD with excellent accuracy. Subsequently, the mRNA levels of the hub ARGs were validated by quantitative real-time PCR (qRT-PCR). Based on differentially expressed ARGs, two aging-related subtypes were identified. Distinct biological functions and immune characteristics including infiltrating immunocytes, immunological reaction gene sets, the human leukocyte antigen (HLA) gene, and immune checkpoints were revealed between the subtypes. Additionally, the black module correlated with subtype-1 was manifested as the hub aging-related module and its latent functions were identified.

**Conclusion:**

Our findings highlight the critical implications of aging-related genes in modulating the immune microenvironment. Four hub ARGs (BLM, FOS, IGFBP3, and PDGFRB) formed a classification model, and accompanied findings revealed the essential role of aging in the immune microenvironment for PD, providing fresh inspiration for PD etiopathogenesis and potential immunotherapy.

## Introduction

Periodontitis (PD) is a considerably prevalent chronic inflammatory disease, which results in irreversible destruction of alveolar bone, causing tooth mobility and drifting (1). While PD is the sixth-most common disease, almost 50% of adults have PD, and 9.8% (796 million people) suffer from its severe form (2, 3). More importantly, epidemiological studies implicate chronic systemic inflammation or infection attributed to PD as an independent risk factor for aging-related diseases such as diabetes mellitus and hypertension (4, 5).

Aging is defined as a time-dependent functional deterioration that generates numerous chronic and age-related pathologies (6). Overwhelming research reported that aging plays vital roles in various diseases such as cardiovascular disease (7, 8), neurodegenerative disorders (9, 10), and tumors (11, 12). In particular, aging affects the maintenance of bone remodeling and metabolism, and the development of an inflammatory environment leading to increased bone resorption (13). Besides, aging influences immune systems including immune-cell function and effector biomolecules, leading to processes reflecting immunosenescence, immunoactivation and inflammaging (14, 15). The coincident loss of immune response capacity with aging, accompanied by chronic low-grade inflammation, can modify immunocompetence and accelerate the pathogenesis of diseases (16). Furthermore, elderly individuals show increased susceptibilities to autoimmune, infectious, and inflammatory diseases, including PD (17, 18).

Data from epidemiological studies have demonstrated that the prevalence of PD increases with aging, which is reflected in an increase of proportion in moderate PD, with only slight changes in mild and severe PD over the 30-80 year age range (19, 20), and the prevalence of severe PD shows an aging-related increase with the peak incidence being observed at young adult age (35-40 years) and remains stable at older ages (21). This indicates that the increased prevalence and severity of PD is not an inevitable consequence of aging. Rather, it is possible the complicated result of altered disease susceptibility and host response associated with aging. Aging alone does not cause severe loss of periodontal attachment in healthy elderly individuals. Instead, the effects of aging on periodontium are based on molecular changes in the periodontal cells, which aggravate the process of PD in elderly patients. Cytologically speaking, evidence showed that cellular senescence, stem cell exhaustion, and immunoaging are hallmarks of biological aging implicated in the breakdown of periodontal homeostasis and the pathophysiology of PD (22). Aging-related genes (ARGs) potentially affect periodontal ligament stem cells (PDLSCs) in a complex way. For instance, Li et al. found that the proliferation, osteogenic/adipogenic/chondrogenic differentiation, and immunosuppressive ability of PDLSCs decreases, whereas apoptosis increases with the aging process (23). Notably, although bacteria are essential to initiate the periodontal inflammatory reaction, the host immune reaction ultimately causes periodontium destruction. Previous studies have revealed that aging promotes pathogenic microbial colonization while arousing a pro-inflammatory microenvironment to exacerbate periodontal inflammation and bone loss (24–26). Tan et al. found that inflamm-aging-related cytokines IL-17 and IFN-γ were associated with alveolar bone resorption in PD (27). A non-human primate study revealed that the levels of inflammatory mediators generally exhibit an age-related increase (28). Results from human studies identified that older individuals have a more severe inflammatory response in an experimental gingivitis model, the gingival lesions from the older individuals contain greater composition of B-cells and a lower density of polymorphonuclear leukocytes (29). However, in-depth knowledge is required to fully uncover ARGs and to expound comprehensive correlations between aging and the PD immune microenvironment, which allows us to develop strategies to maintain good oral health in the aging population and to decrease the burden of PD.

**Figure 1:**
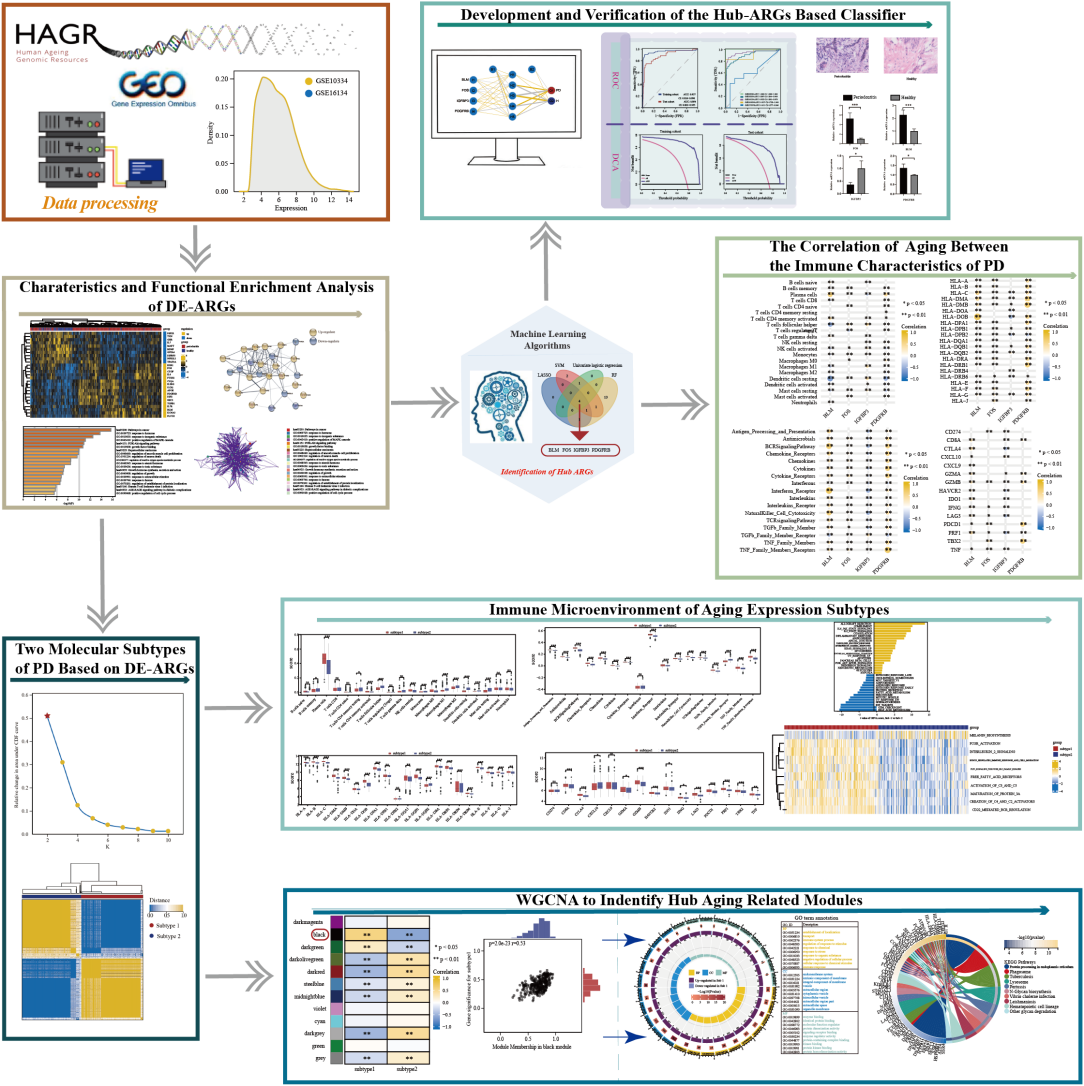
The work flowchart of the study (*p < 0.05, **p < 0.01, ***p < 0.001, and ns means not significant).

Up to now, research about aging is mainly about its influence in the tumor immune microenvironment (12). Similarly, the PD immune microenvironment could be defined as the cellular phenotypes, immune-related pathways, and immune-related markers that are known to be affected by infiltrated immune cells in the interstitial space of periodontium and by the interactions between different cell types (30). Accordingly, it is reasonable to hypothesize that aging must own substantial effects on regulating the PD immune microenvironment. In the present study, a series of bioinformatics approaches were employed to excavate and screen hub candidate ARGs and to reveal the relationship between PD immune microenvironment and aging, followed by a flow chart of the study (**Figure 1**). Our findings provide further insight into the role of ARGs in the PD immune microenvironment. We hope that the classification model based on the hub ARGs could reveal new molecular mechanisms of how aging acts on PD, and improve the early diagnosis and immunotherapy of PD.

## Methods and materials

### Data Collection and preprocessing

A total of five datasets, namely GSE16134-GPL570 (31), GSE10334-GPL570 (32), GSE106090-GPL21827 (33), GSE173078-GPL20301 (34), and GSE23586-GPL21827 (35), were downloaded from the Gene Expression Omnibus (GEO) database (https://www.ncbi.nlm.nih.gov/geo/). More details of the collected datasets were presented in **Table S1**. Probes were annotated as the gene symbols. Probes without matching gene symbols and matching multiple symbols were excluded. The gene expression value of duplicate gene symbol was calculated as the median value. The GSE16134 and GSE10334 dataset were merged using the R package “inSilicoMerging” (36), and then the batch effects were removed *via* ComBat method in the R package “sva” (37) and finally the merging was randomly split at a 7:3 ratio into a training cohort and a testing cohort. The GSE106090, GSE173078, and GSE23586 datasets were deployed as the external validation datasets. A total of 307 ARGs were obtained from Human Aging Genomic Resources (HAGR) (https://genomics.senescence.info/genes/index.html) (38), which were listed in **Table S2**.

### Identification and functional enrichment analysis of differentially expressed ARGs (DE-ARGs)

The criteria were formulated as |log_2_fold-change (FC)|>0.5 and p-value <0.05 to filter out differentially expressed genes (DEGs) using the R package “limma” (39). Twenty-seven DE-ARGs were identified under the intersection of 1251 DEGs and 307 ARGs from HAGR. A volcano plot and heatmap were used to visualize the DE-ARGs using the R package “ggplot2” and “pheatmap”, respectively. Principal component analysis (PCA) was performed on the DE-ARGs using the R package “factoextra” (https://cloud.r-project.org/package=factoextra/) to show the clustering of samples with the first two components. Subsequently, a protein-protein interaction (PPI) network was constructed to assess the gene interactions among the DE-ARGs using Search Tool for the Retrieval of Interacting Genes (STRING, version 11.5, https://cn.string-db.org/) database (40) with a confidence score >0.4 as the cut-off criterion, visualized with Cytoscape software, V3.8.2 (41). Functional and pathway enrichment analysis including Gene Ontology (GO) terms and Kyoto Encyclopedia of Genes and Genomes (KEGG) pathway analysis were conducted on the DE-ARGs using Metascape (https://metascape.org/gp/index.html). Based on their membership similarities, terms with a p-value <0.01, a minimum count of three, and an enrichment factor >1.5 were collected and grouped into clusters.

### Identification of hub ARGs by integrating four machine learning algorithms

The Least Absolute Shrinkage and Selection Operator (LASSO) logistic regression, Support Vector Machine-Recursive Feature Elimination (SVM-RFE), and Random Forest (RF) algorithms were employed independently to screen out the candidate hub ARGs *via* the R package “glmnet” (42), “e1071” (43) and “randomForest” (44), respectively. As for LASSO, 10 cross-validation was performed to screen the optimal tuning parameter (λ). SVM is a monitored machine learning technology extensively used for categorization and regressive analysis. An RFE arithmetic was utilized to screen the optimum genes from the training cohort for avoiding overfit. Hence SVM-RFE was utilized to identify the gene set with the greatest discrimination ability. RF analysis is an appropriate approach with the benefits of no limits on variable conditions and better accuracy, sensitivity, and specificity. Simultaneously, univariate logistic regression was also implemented to identify the hub ARGs. Ultimately, genes that overlapped among the four machine learning algorithms were defined as the hub ARGs.

### Construction and verification of an artificial neural network model based on PD-related hub ARGs

ANN is a main part of deep learning affiliated to artificial intelligence. The training cohort was used to construct an ANN model using the R package “neuralnet” (45). The processed training data were input into the ANN model; four input layers, five hidden layers, and two output layers were set for the ANN. The R package “Caret” (46) was employed to perform 5-fold cross-validation on the ANN model to reduce overfitting and optimize the model. Besides, receiver operating characteristic (ROC) (47) and decision curve analysis (DCA) (48) were utilized for the robustness and clinical significance of the ANN model. Subsequently, a hub-ARGs based classification model, which was also known as “classifier” in this study, was verified in the test cohort, as well as the external validation datasets by the same token.

### Human sample collection and specimen histology

The specimen of gingival tissue collection was divided into two groups. The control group included four healthy gingival tissues from four patients, which were required for crown lengthening surgery, and signed informed consent to participate in this experiment. The PD group was four gingival tissues with PD lesions from four patients, which were diagnosed as severe PD and required periodontal flap surgery and signed informed consent to participate in the experiment. All gingival tissue sizes were about 2 mm^3^. After excision, the tissues were immediately placed in an RNA preservative reagent (RNAlater, Invitrogen), and then stored at 4°C overnight. Subsequently, the tissues were stored at −80°C until RNA extraction. **Table S3** presents the clinical information including gender, age, and exclusion criteria on the eight patients. As for specimen histology, gingival tissues obtained from healthy patients and PD patients were sequentially fixed in 4% buffered formalin for 48 h, dehydrated in graded ethanol, embedded in paraffin, and cut into 5 μm sections. The sections were stained with hematoxylin-eosin (HE) for histological analysis.

### RNA extraction and quantitative real-time PCR

The total RNA of 4 pairs of healthy and PD gingival tissues (control group n = 4; PD group n = 4) from 8 patients was extracted by RNAiso plus kit (TaKaRa) and reversely transcripted to cDNA. qRT-PCR was carried out using SYBR Premix Ex Taq (TaKaRa). The relative mRNA expression was calculated using the 2^△△CT^ method normalized to the level of GAPDH. The primers of four hub ARGs and the internal control gene are listed in **Table S4**.

### Correlation analysis between hub ARGs and immune characteristics

Evaluation of putative immunocyte proportion of gingival tissues with 1,000 iterations was calculated by using the Cell-type Identification By Estimating Relative Subsets Of RNA Transcripts (CIBERSORT). Samples possessing a CIBERSORT p-value <0.05 were selected for further analysis (49) to ensure the reliability of the deconvolution algorithm. Immunological pathway activities were investigated *via* single-sample gene set enrichment analysis (ssGSEA) (50). The preceding study provided the verified leukocyte gene signature set (LM22) for CIBERSORT (51). The gene sets of immunological pathways were acquired from the ImmPort database (http://www.immport.org) (52). And the immune checkpoints were extracted from previous literature (53). The relative abundance of immunocytes, the enrichment scores of immune reactions, the status of the HLA gene, and immune checkpoint in PD and healthy samples were examined by the Wilcoxon test. Subsequently, the correlation coefficients of the putative immunocytes proportion, the immunological pathways enrichment scores, the HLA gene, and immune checkpoint expression values between hub ARGs were calculated by Spearman correlation analysis.

### Identification and functional enrichment analysis of aging expression subtypes

Unsupervised clustering analysis was applied to single out distinctive aging expression subtypes based on 27 DE-ARGs expression profiles. To control the robustness of the clustering, 1000 iterations were performed, and each iteration contained 80% of the samples. The R package “ConsensuClusterPlus” was conducted to implement the above process (54). The cumulative distribution function (CDF) curve of the consensus score was used to define the optimal cluster number. The gene distribution of subtypes was evaluated by PCA. The Wilcox test was performed to examine the expression status of the ARGs between the two distinct aging-related subtypes. Gene-set variation analysis (GSVA) algorithm was employed to investigate HALLMARKS and Reactome pathways of each subtype (55).

### Identification of aging phenotype-related gene modules

Ranked by the sum of expression values, the top 5000 mRNAs were selected as the genes of interest. Aging phenotype-related genes that take part in immunity were screened out by taking the intersection of the immune-related genes from the ImmPort database and the 5000 genes. Then, the R package “WGCNA” was employed to detect genes in the modules associated with aging phenotypes (56, 57). After determining the power of 9 and a minimum size (Gene group) of 30 for the genes dendrogram, the R package “ggplot2” was used to visualize the correlations between module eigengenes and aging phenotype-related patterns. Afterwards, the module with the highest correlation coefficients and the most significant p-value was determined as the key module, and genes in the key module were defined as aging phenotype-related genes. Furthermore, functional enrichment analysis was performed on these genes by the R package “ClusterProfiler” to identify GO and KEGG pathways.

## Results

### Identification of ARGs and enrichment analysis

**Figure 2:**
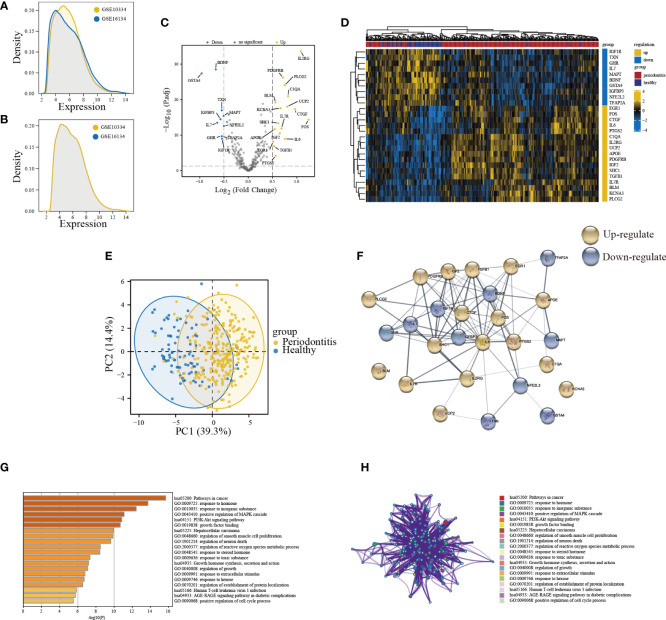
Expression patterns and biological significance of differentially expressed aging-related genes (DE-ARGs) in PD. **(A, B)** The density of the merge of GSE10334 and GSE16134. **(C)** Volcano plots visualizing the expression patterns of DE-ARGs in PD and healthy samples. **(D)** Heatmap of the 27 DE-ARGs in PD and healthy samples. **(E)** Principal component analysis of the training cohort based on dysregulated aging-related genes. **(F)** PPI network of proteins encoded by DE-ARGs through the STRING database. **(G)** Bar graph of enriched terms. The bar was colored by values of p. The lower the values of p, the deeper the color. **(H)** The network of enriched terms. The top 20 clusters were selected and rendered as a network, in which terms with a similarity score > 0.3 are connected by an edge. The thickness of the edge represents the similarity score.

The sample distribution of each dataset was observed to be totally different before removing the batch effect, indicating that there was a batch effect (**Figure 2A**). After removing the batch effect, the data distribution converged (**Figure 2B**). A total of 27 DE-ARGs were identified including 17 upregulated and 10 downregulated ARGs (**Figure 2C**; **Table S5**), and were illustrated by the heatmap in **Figure 2D**. Then, the PCA algorithm was used to analyze the training cohort, showing that the PD and healthy samples could be well distinguished by these DE-ARGs (**Figure 2E**). The PPI network showed the intricate relevance of ARGs-associated proteins (**Figure 2F**). A bar diagram (**Figure 2G**) and corresponding network (**Figure 2H**) were depicted to show the results of Metascape analysis: spots represented functions or pathways, yet larger and connected points represented the presence of more similar genes between the functions or pathways. Enriched functional GO terms, including biological processes (BP), cellular components (CC), and molecular functions (MF), as well as KEGG pathway analysis of DE-ARGs were performed to explore their biological functions. Terms associated with inflammation and immune function were the most enriched, such as pathways in cancer, response to hormone, positive regulation of MAPK cascade, and PI3K-Akt signaling pathway.

### Development and verification of the hub-ARGs based classifier *via* multiple machine learning algorithms and ANN model

**Figure 3:**
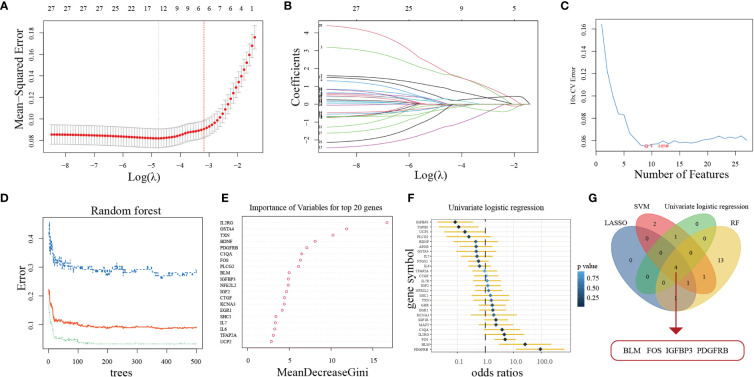
Four machine learning algorithms were used for hub ARGs. **(A)** The least absolute shrinkage and selection operator (LASSO) coefficient profiles of the 27 DE-ARGs. **(B)** 10-fold cross-validation for optimum tuning parameter (λ) selection using LASSO. **(C)** Estimating 10-fold cross-validation error using the support vector machine recursive feature elimination (SVM-RFE). **(D)** The relationship between the number of decision tree and the model error. **(E)** Random Forest (RF) algorithm showed the top 20 candidate genes. **(F)** Univariate logistic regression analysis results. **(G)** Venn diagram showed that four hub ARGs are identified *via* the above four algorithms.

Aimed at constructing a hub-ARGs based classifier that can accurately distinguish PD from healthy controls, four machine learning algorithms including the LASSO regression algorithm (**Figure 3A, B**), SVM-RFE algorithm (**Figure 3C**), RF algorithm (**Figure 3D, E**), and univariate logistic regression analysis (**Figure 3F**) were implemented on the 27 DE-ARGs to select candidate hub ARGs, respectively. The genes obtained by the four algorithms were overlapped, and finally, four hub ARGs (BLM, FOS, IGFBP3, and PDGFRB) were identified (**Figure 3G**).

**Figure 4:**
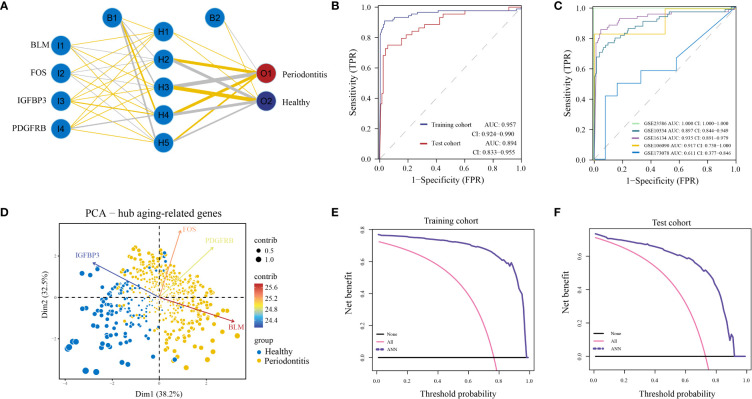
Construction of artificial neural network (ANN) model. **(A)** ANN model has four inputs, five hidden neurons, and two outputs. In this case, the 4 inputs represent the category values of the four hub ARGs. **(B)** The ROC curve of the ANN model in the training cohort and test cohort. **(C)** The ROC curve of the ANN model in validation cohorts including GSE16134, GSE10334, GSE106090, GSE173078, and GSE23586. **(D)** Principal component analysis (PCA) of four hub ARGs between healthy and periodontitis, the contribution of each gene is represented by a colorful arrow. DCA results to evaluate the clinical value of the ANN model *via* training cohort **(E)** and test cohort **(F)**.

Finally, we built an ANN model for classifying gene expression data between PD and control samples based on the four hub ARGs (**Figure 4A**; **Table S6**). ROC curve proved that our predictive model, the classifier based on hub ARGs, was very reliable, with an AUC of 0.957 in the training cohort and an AUC of 0.894 in the testing cohort (**Figure 4B**). Furthermore, the AUC values of the validation datasets-GSE23586, GSE10334, GSE16134, GSE106090 and GSE173078 were 1.000, 0.897, 0.935, 0.917 and 0.611, respectively (**Figure 4C**), which confirmed the high validity of the classifier. PCA showed that PD and healthy samples could be well distinguished by the four hub DE-ARGs with the ANN model (**Figure 4D**). Furthermore, the DCA curve was plotted to test the clinical influence of the ANN model in training (**Figure 4E**) and testing (**Figure 4F**) cohort.

### Quantitative real-time PCR validation

**Figure 5:**
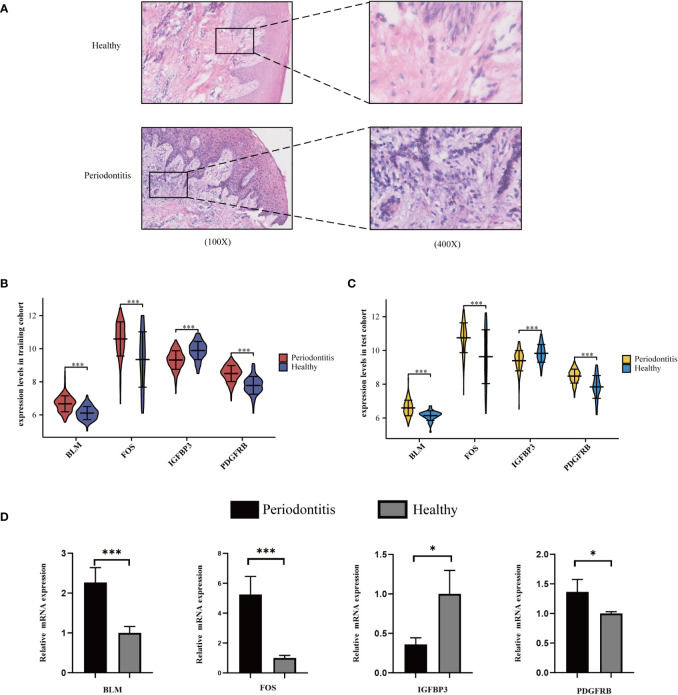
Validation of hub ARGs. **(A)** Representative images of HE staining of human periodontal tissues. **(B)** The expression status of the four hub ARGs was presented in the form of a violin diagram in the training cohort and **(C)** test cohort. **(D)** The qRT-PCR validation of the four hub ARGs (*p< .05; ***p< .001).

We found that inflammatory cell infiltration into gingival tissue is more extensive in the PD group than in healthy controls (**Figure 5A**). The expression level of the four hub ARGs was represented in the form of a violin diagram in the training cohort (**Figure 5B**) and test cohort (**Figure 5C**). Besides, the results of qRT-PCR for the four hub mRNAs were presented (**Figure 5D**). In the healthy and PD comparison, the expression levels of BLM, FOS, and PDGFRB are higher in the PD group, while IGFBP3 is lower. The results of qRT-PCR were consistent with the bioinformatics analyses; thus, the reliability of the ANN model was confirmed.

### The PD immune microenvironment and aging were correlated

**Figure 6:**
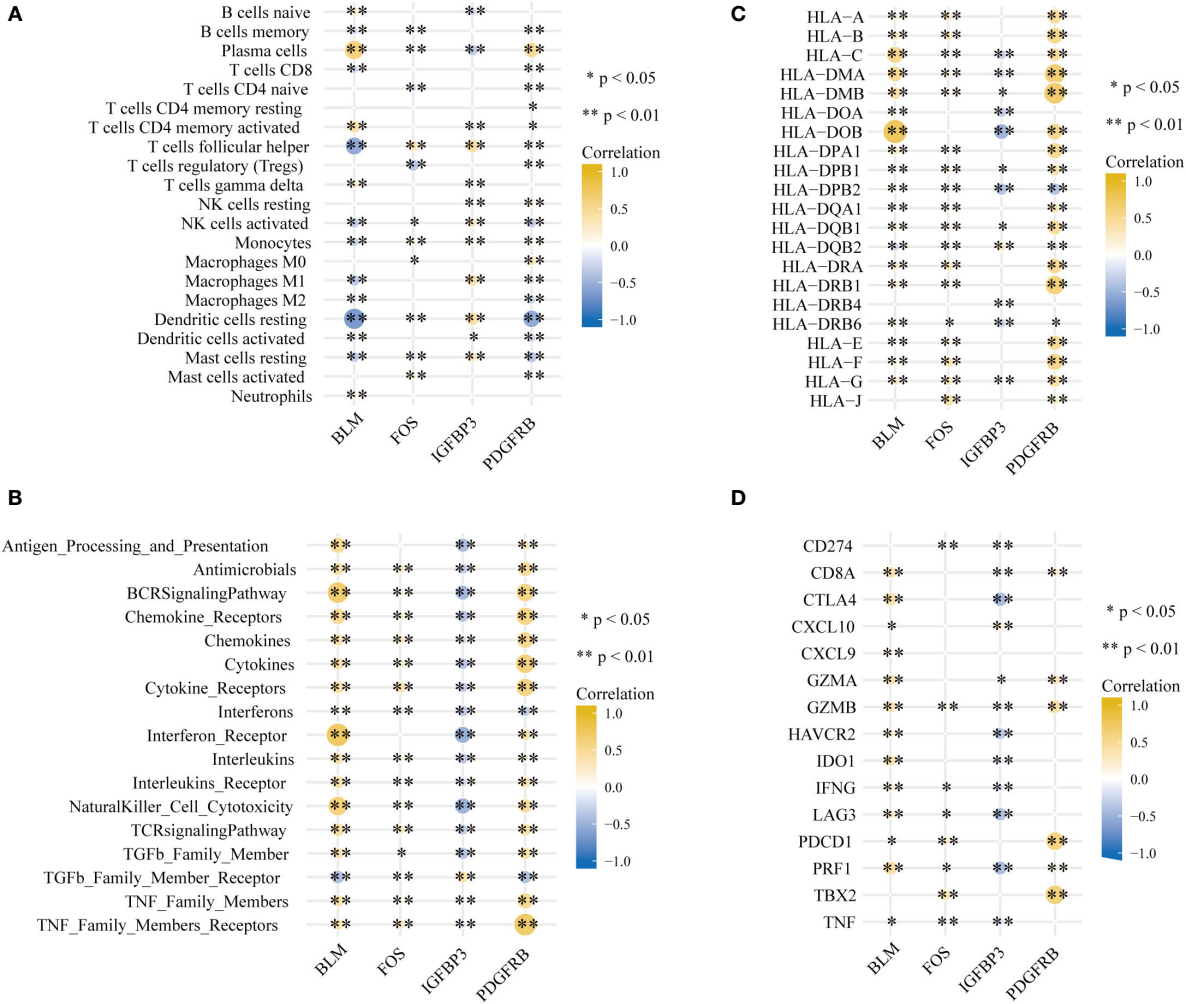
Signatures associated with immune infiltrations in PD. **(A)** Correlation coefficients between the immune cell infiltrations, **(B)** the enrichment scores of immune reaction, **(C)** the expression of HLA gene and **(D)** immune checkpoints and four hub aging-related genes (*p < 0.05, **p < 0.01).

It is worth mentioning that the immune microenvironment played significant roles in the pathogenesis of PD and was connected closely with aging. Hence characteristics of the PD immune microenvironment were described to further comprehend the underlying mechanisms. CIBERSORT was employed for the estimated infiltration of immunocytes in each sample, while more plasma cells were evaluated in PD samples. Eosinophil was not detected from the CIBERSORT result (**Figure S1A, B**). Higher activation of immune reactions, such as interferon receptor and BCR signaling pathway (**Figure S1C**), higher expression level of HLA genes, such as HLA-A, HLA-B, and HLA-C (**Figure S1D**) and higher expression level of immune checkpoints, such as GZMB and CD8A (**Figure S1E**) were detected in PD compared to healthy controls. Correlation analysis found that the most positively correlated immunocyte aging gene couple is BLM-Plasma cell, whereas BLM-Resting dendritic cell is the most inversely correlated couple (**Figure 6A**). Moreover, we noticed that BLM and PDGFRB have the most significantly correlated immunological reaction gene sets, with BLM being positively related to interferon receptor and BCR signaling pathway, and PDGFRB being positively related to TNF family members receptors (**Figure 6B**). This indicated BLM and PDGFRB played vital roles in the above-mentioned reactions in PD. Similar trends were observed in HLA genes as well, for example, BLM-HLA_DOB is the most positively correlated pair, and PDGFRB-HLA_DMA, as well as PDGFRB-HLA_DMB, are the two most positively correlated pairs (**Figure 6C**). Concerning immune checkpoints, PDGFRB-TBX2 is the most positively correlated pair, while IGFBP3 is negatively correlated with CTLA4, LAG3, and PRF1(**Figure 6D**).

### Two aging expression subtypes were determined based on the 27 DE-ARGs

**Figure 7:**
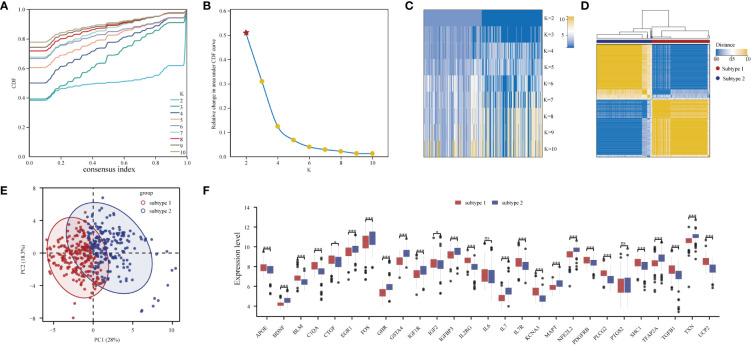
Unsupervised clustering of 27 DE-ARGs. Identifying two distinct ARG-subtypes in PD. **(A)** Consensus clustering cumulative distribution function (CDF) for k = 2- 10. **(B)** Relative change in area under CDF curve for k = 2- 10. **(C)** Heatmap of k with two subtypes in periodontitis. **(D)** Heatmap exhibiting the two subtypes of PD samples with k =2. **(E)** Principal component analysis for the transcriptome profiles of the two distinct subtypes, showing a remarkable difference in transcriptome between different subtypes. **(F)** The expression status of 27 DE-ARGs in the two subtypes (*p < 0.05, ***p < 0.001, and ns means not significant).

Through consensus clustering analysis, PD samples were clustered into two molecular subtypes based on the expression profiling of the 27 DE-ARGs (**Figures 7A–D**; **Table S7**). PCA algorithm indicated a remarkable difference between the two subtypes (**Figure 7E**). Subsequently, the expression status of the DE-ARGs was compared, and the vast majority of the genes prominently varied (**Figure 7F**).

### Different immunological features were detected in the aging expression subtypes

**Figure 8:**
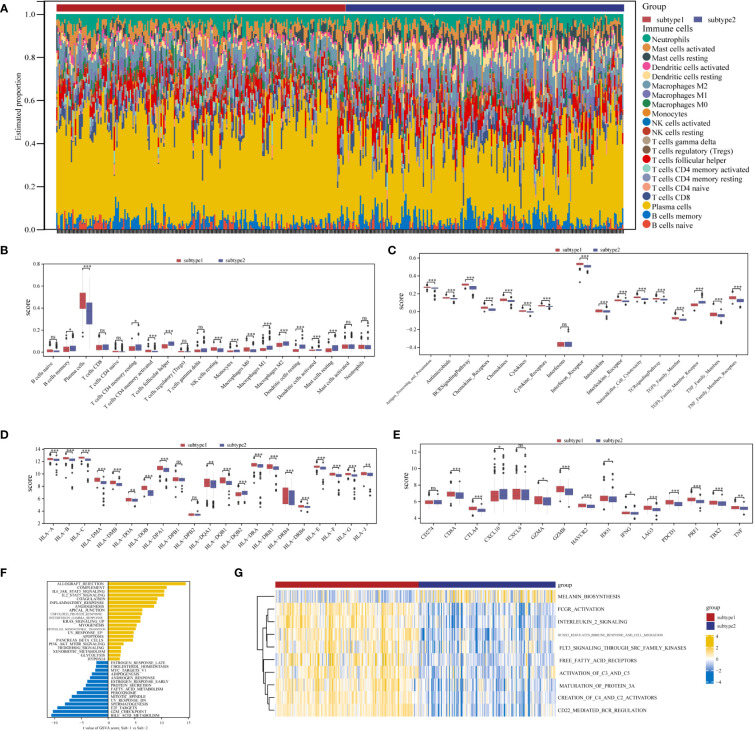
Diversity of immune microenvironment characteristics between two distinct ARG-subtypes in PD. **(A)** The proportion of cell infiltration in 22 types of immune cells in the two subtypes. **(B)** Box plots showing the differences in immune cell infiltrations in the two distinct subtypes. **(C-E)** The activity differences of each immune reaction gene set, the expression differences of each HLA gene, and the expression differences of each immune checkpoint in the two distinct subtypes, respectively (*p < 0.05, **p < 0.01, ***p < 0.001, and ns means not significant). **(F, G)** The underlying biological function characteristics diversity between the distinct subtypes, and the differences of the HALLMARKS pathway and Reactome pathway enrichment score between subtype-1 and subtype-2 (**F** for the HALLMARKS pathway and **G** for the Reactome pathway).

Marked differences were noted in the immunological features between the two aging expression subtypes, indicating a close relationship between aging and immune regulation. CIBERSORT was applied for estimated infiltration of immunocytes in each subtype revealing significant differences. Eosinophil was not detected from the CIBERSORT result (**Figure 8A**). Immunocytes differ between the two subtypes (**Figure 8B**): subtype-1 has relatively low infiltrated immunocytes compared with subtype-2, while more plasma cells are evaluated in subtype-1. As demonstrated in **Figure 8C**, subtype-1 has more active immune reactions than subtype-2, and interferon receptor is the most active one. Similar trends are observed in HLA genes and immune checkpoints (**Figures 8D, E**). These results indicated that subtype-1 led to a distinctly active immune response while subtype-2 mediates a mild immune response in PD, and proved that aging-related subtypes played vital roles in shaping different immune microenvironment characteristics in PD.

### Biological characteristics of the aging expression subtypes

**Figure 9:**
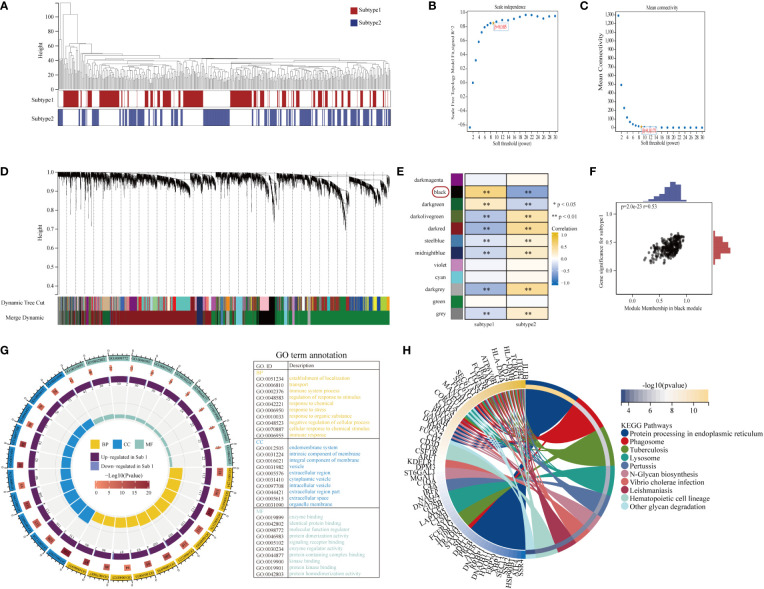
Construction and module analysis of weighted gene co-expression network analysis (WGCNA). **(A)** Sample dendrogram and trait heatmap. **(B, C)** The selection of the smallest soft threshold. **(D)** Gene dendrogram obtained by average linkage hierarchical clustering. The color row underneath the dendrogram showed the module assignment determined by the Dynamic Tree Cut. **(E)** Heatmap of the correlation between module eigengenes and the aging modification patterns. **(F)** A scatterplot of gene significance (GS) for subtype-1 vs module membership (MM) in the black module. GS and MM exhibited a very significant correlation, implying that hub genes of the black module also tend to be highly correlated with subtype-1. **(G)** Gene Ontology enrichment analysis of aging phenotype-related genes, the outermost ring represented the name of pathways. The second outer ring represented the number of genes in pathways, and the heights of the columns in the inner ring indicated the value of GeneRatio. **(H)** Chord plot depicting the relationship between genes and KEGG signaling pathways.

To investigate other distinct biological functions between the subtypes, the GSVA algorithm was employed to calculate the enrichment scores of the Reactome and HALLMARKS pathways of the subtypes (**Figure 8F** for the HALLMARKS pathway and **Figure 8G** for the Reactome pathway). We discovered that subtype-1 has more enriched pathways, while the famous inflammation pathways such as IL6-JAK-STAT3, IL2-STAT5, and PI3K-AKT-MTOR prominently vary. Besides, KEGG pathways remarkably differ among the two subtypes and enrich in pathways such as melanin biosynthesis, fcgr activation, interleukin 2 signaling, and runx3 regulates immune response and cell migration. Next, to further comprehend the molecular mechanisms by which genes are involved in aging-mediated regulations, 301 aging phenotype-related genes that take part in immunity were screened out (**Table S8**). Furthermore, to identify hub ARGs modules, 12 gene modules were determined based on a dynamic tree utilizing WGCNA (**Figures 9A–E**; **Table S9**). Based on the module–trait relationships between 12 modules and the two subtypes, the most significant correlation was seen between the black module. The association of the module membership in the black module with gene significance in subtype-1 was visualized in the scatter plots (**Figure 9F**). Subsequently, the GO and KEGG pathways were investigated to explore the functional mechanisms indicated by the aging-mediated black gene network module. Most genes in the black module were found to be enriched in BPs such as establishment of localization, transport, and immune system process, CCs such as endomembrane system and intrinsic component of membrane, and MFs such as enzyme binding and identical protein binding (**Figure 9G**). The KEGG pathway analysis revealed that the black module genes were mainly enriched in pathways such as protein processing in endoplasmic reticulum, phagosome, and lysosome (**Figure 9H**).

## Discussion

As evidence accumulates worldwide, PD is a chronic inflammatory disease that involves complex interactions between pathogens and immune reactions (1). Unequivocally, the aging process contributes to the incidence and severity of PD and has been concerned with altered physiology that leads to dysregulation of the immune system to the noxious challenge of the disease-associated oral microbiome (24). Therefore, potential biomarkers for early diagnosis and therapeutic targets are in sore need. Accumulating evidence confirmed the indispensable role of aging in both innate and adaptive immune reactions (58–60). It is reasonable to suspect that aging must own substantial effects on regulating the PD immune microenvironment. In the present study, with the availability of gene expression information in public databases, multiple comprehensive bioinformatics analyses were employed to describe many molecular aspects of aging in the pathogenesis of PD.

Primarily, twenty-seven DE-ARGs were found in PD. The DE-ARGs correlated and interacted with each other, revealing a regulatory network of aging in PD. According to functional and pathway enrichment analysis, the DE-ARGs were considered to be prominently abundant in inflammation- and immunity-related processes such as pathways in cancer, response to hormone, positive regulation of MAPK cascade, and PI3K-Akt signaling pathway. Some of the pathways present here were consistent with previous studies. Many proinflammatory pathways in PD are linked to carcinogenesis (61). For example, porphyromonas gingivalis (P. gingivalis) is found to be associated directly with the oncogenic pathways through the activation of the PI3K-Akt signaling pathway (62). Konstantonis et al. reported that by activating ERK, JNK, and p38 MAPK, senescent PDLSCs result in cell differentiation when suffer from cyclic mechanical deformation (63). Regarding the pathway response to hormone, sex steroids such as estrogens and androgens are fundamental to bone homeostasis and immune function. Age-associated reductions in sex steroids cause obvious temporal increasing susceptibility to PD, particularly among women with estrogen deficiency during perimenopause (64). Recent research has investigated that postmenopausal women treated with estrogen for osteoporosis have a lower prevalence of severe PD than women in a control group not receiving such therapy (65). This case reminds us of how a medical intervention may have secondary benefits on periodontal conditions and reduce complications with aging. Sex-specific temporal differences in gene regulation, particularly in aging, have profound influences on disease susceptibility (66). In general, women generate more robust and potentially protective humoral and cell-mediated immune responses, whereas men frequently elevate a more aggressive and potentially damaging inflammatory immune response to microbial stimuli (67, 68), perhaps accounting for an increased prevalence and severity of PD in men, as multiple epidemiologic studies showed (19, 69, 70). This indicates that sex differences in the pathway response to hormone associated with aging may have the potential correlation to the immune microenvironment for PD. However, sex effects on the prevalence/extent/severity of PD were not taken into account in the current study, which is a limitation. The enriched pathways mentioned above may provide important insight into aging and into the onset, progression, and therapeutic outcomes of PD.

More interestingly, our study identified four hub ARGs (BLM, FOS, IGFBP3, and PDGFRB) by integrating four machine learning algorithms, and revealed a hub-ARG based classifier that can well distinguish PD from healthy controls by ANN model. In other words, the classifier might provide oral clinicians an experimental strategy to distinguish patients with high risk of PD from healthy patients beforehand, to intercept biological aging when still “subclinical” and formulate interventions for halting or delaying the trajectory toward PD while patients are still chronologically young. BLM, a kind of RecQ-like helicases, plays vital roles in maintaining genome integrity. Defects in BLM are associated with the Blooms syndrome, an autosomal recessive disorder featured by chromosome gaps and breaks, elevated sister chromatid exchange, mitotic hyper-recombination, as well as aberrant DNA replication events (71). But experimental evidence about the relationship between BLM and aging in PD is lacking. The proto-oncogene FOS (also named C-Fos) is found to be involved in the transcriptional regulation of collagenase and cell proliferation genes in periodontal gingival fibroblasts (72). Besides, FOS acts as an osteoclastogenic marker to participate in the progress of PD mediated by inflamm-aging-related cytokines *in vitro* and *in vivo (* 27). A deep learning-based autoencoder predicts FOS to be critical immunosuppression genes and mediate immune suppression in PD (73). IGFBP3, the sole downregulated hub ARGs in our study, represented the main binding protein that is widely distributed in the serum, tissue, and extravascular fluid. Experimentally evidence found an inverse correlation of serum IGFBP3 levels with clinical attachment loss and number of missing teeth, predicting IGFBP3 to be associated with the severity of PD (74). Moreover, given the facts that the levels of IGFBP3 decrease throughout lifetime (75) and PDLSCs, cementum, and dentine may serve as local reservoirs for IGFBP3 (76), we might speculate that the degradation of the levels of IGFBP3 with age could conduce to PD. PDGFRB is a dimeric receptor tyrosine kinase that plays critical roles in cell growth, survival, and differentiation (77). Numerous studies investigated the role of PDGFRB in the development of Alzheimer’s disease (78, 79). However, there was no report about PDGFRB and PD. The application of ANN in constructing disease models has proven to be sophisticated (80). The outstanding novelty of our study was firstly employed ANN model to construct a classifier based on the four hub ARGs. The results of ROC and DCA showed that the classifier for PD achieved excellent accuracy in disease classification and laid the groundwork for future molecular mechanisms research.

To explore the PD immune microenvironment, ssGSEA and CIBERSORT algorithms were employed to calculate the immunocytes composition and the immune reaction activity; the expression of the HLA gene and immune checkpoints were analyzed as well. More plasma cells were evaluated in PD samples. Results from animal studies have revealed more plasma cells in gingival tissue samples from older dogs (81). It was reported that breaching the balance among microbiome invasion, host defense, and tissue regeneration may cause plasma cell–induced pathologic bone resorption, leading to an inadequate horizontal/vertical bone volume that ultimately results in tooth loss (82). This finding suggested that plasma cell infiltration played a significant role in host defense against PD. Higher activation of immune reactions, higher expression level of the HLA gene and immune checkpoints were observed in PD. Then the correlation between immune characteristics and hub ARGs was explored. Among the ARGs pairs, BLM and PDGFRB showed the strongest positive correlation with immune characteristics in PD. For instance, BLM, as well as PDGFRB, were positively and negatively correlated with plasma cells and dendritic cells resting, respectively. It is reported that aging appears to functionally impair dendritic cell uptake of antigens, phagocytosis of apoptotic cells, and migration and priming of both CD4^+^ and CD8^+^ T cells (83). Besides, BLM was positively correlated with BCR signaling pathway and HLA-DOB, and PDGFRB was positively correlated with TNF family members receptors and the immune checkpoint TBX2. BLM is related to immune system, connecting DNA damage to cellular innate immune response (84). Kassambara et al. had experimentally validated a potent role for BLM in regulating cell survival and proliferation during plasma cell differentiation (85). Dendritic cells are the most effective specific antigen-presenting cells. Li et al. noted that PDGFRB is exclusively expressed in Lymph node fibroblastic reticular cells (FRCs) and depleting FRC-ECM (extracellular matrix) laminin α4 causes reduction in Tregs and dendritic cells (86). Additionally, a recent study suggested that BCR signaling pathway may be involved in PD with Down syndrome (87). Predominantly, with the rapid emergence of molecular targeted therapies, there has been increasingly more attention on immune checkpoints, the critical regulators of immune homeostasis. The targeting of the PD-1/PD-L1 immune checkpoint could be considered an appropriate approach to improve the treatment of PD (88). However, there is negligible data available regarding the relationship between PD and immune checkpoint TBX2 mentioned in our result. Taken together, these findings may point out the direction of the aging immune regulation mechanism and offer novel prospects for further exploration of aging in PD.

To further explore the mechanistic aspects of immune characteristics of PD, unsupervised clustering of the PD samples based upon DE-ARG expression profiles was applied and demonstrated two distinct ARGs subtypes, indicating that aging played a potent impact on the PD immune microenvironment. The modification of subtype-1 has more active immune reactions and higher expression levels of HLA genes and immune checkpoints than subtype-2. For example, subtype-1 has more activation in the famous signaling pathway of IL6-JAK-STAT3, IL2-STAT5, and PI3K-AKT-MTOR. The mammalian target of rapamycin (mTOR) is considered a pivotal enzyme at the crossroad of nutrient sensing and cell growth, and proper activation of mTOR signaling pathways is essential for healthy aging. Rapamycin, a kind of drug approved by the Food and Drug Administration, directly targets mTOR, exhibiting the capacity to ameliorate age-related phenotypes and prolong life span (89). Short-term treatment with rapamycin in aged mice demonstrated its effectiveness in reversing alveolar bone loss (90), and rapamycin suppressed the elevated RANK/OPG ratio characteristic of promoted-resorptive aged bone physiology after eight weeks, possibly providing the basis for oral cavity rejuvenation strategies. Furthermore, other senolytic agents such as MCC950 and some natural compounds have been tested to treat senescence as pharmaceutical interventions (22). However, clinical trials have only recently started, and their safety and efficacy remain to be determined. Considering the varied immune characteristics of each subtype, it confirmed the reliability of our classification of immune phenotypes for the DE-ARGs. This classification strategy for immune subtype can help us understand the underlying mechanism of immune regulation so that precise therapeutic methods can be applied and PD can be subtyped from the molecular level or immune level not only the clinical phenotype level (91). Therefore, aging expression subtypes of gingival tissues can indeed be regarded as an alternative pathobiology-based classification of PD. The potential relevance of these differences in aging mechanisms between subtypes may represent the molecular patterns of PD associated with aging and further exploration in experimental research is warranted.

WGCNA showed a high correlation of the black module with subtype-1, which was found to be enriched in pathways such as protein processing in endoplasmic reticulum, phagosome, and lysosome. As previously described, the endoplasmic reticulum (ER) functions in protein biosynthesis and folding. Increased physiological demand for protein folding can lead to the accumulation of misfolded or unfolded proteins in the ER lumen, a state called ER stress (92), which is the cell response by excitation of the unfolded protein response (UPR) pathway in diverse conditions such as infection and aging (93). The prolonged inflammation of PD triggers UPR, causing decreased osteogenic differentiation of PDLSCs both *in vivo* and *vitro* (94). Others have investigated that the ER stress-induced alveolar bone resorption in PD was independent of inflammatory cytokine release (95). Collectively, these data suggest that multiple pathways of aging may be implicated in PD with different modules; furthermore, distinct molecular subtypes may exist in terms of their relative dominance; eventually, revealing their biological function can conduce to the illustration of PD pathogenesis from the aging regulation perspective.

To the best of our knowledge, our study was the first to construct a hub-ARGs based classifier for discriminating PD from healthy controls, and systematically investigate the correlation between aging and the PD immune microenvironment, which could well enlighten the further immunotherapeutic approaches to improve PD treatment. PD along with other common chronic inflammatory diseases share a relatively small set of common modifiable risk factors, which can lead to increases in systemic inflammation markers and modify gene regulation *via* various biologic mechanisms (96). Intriguingly, such research assumes a profound impact considering the possibility of behavioral interventions and pharmaceutical interventions directed at healthy aging (22), providing a promising management modality for PD and oral-systemic diseases. However, we must admit that this study has some shortcomings. Our study mainly focused on bioinformatics analysis, and most results remain to be confirmed by experiments *in vitro* and *in vivo*; although the four hub ARGs were verified by qRT-PCR, the human gingival sample size is relatively insufficient, which could potentially account for bias, and the larger sample size is required in future studies; the specific mechanism of these four genes in the PD immune microenvironment remains unclear, and, as a result, further experimental studies are necessary for the elucidation of the potential biological mechanisms; the measurements for immune cells and pathway activation are based on the GSVA score, which is calculated by gene expression at the transcriptomic level, which can barely reflect the changes occurring on the protein level; since the clinical information such as the severity, stage or grade of PD was not given in the main datasets GSE16134 and GSE10334, our bioinformatics analyses did not contain the items of the timing for lesion formation. Therefore, future studies are needed to address metadata regarding clinical disease characteristics and their correlation with aging-based subtypes, to explore the dynamics of the timing for periodontal lesion formation and detection, and resolution that could be substantively affected by aging. Nevertheless, the findings furnish a unique platform for exploring the interface of aging-related pathogenesis of PD and offer a new reference for potential immunotherapy targets.

## Conclusion

The present study constructed a “classifier” for PD based on the four hub ARGs (BLM, FOS, IGFBP3, and PDGFRB). Furthermore, two distinct aging-related subtypes were identified, with differences in enriched functional biological functions and immune microenvironment indicated by Infiltrating immunocytes, immunological reaction gene sets, HLA genes, and immune checkpoints. A hub aging-related module of subtype-1was associated with ER and related functions. These findings revealed the underlying regulation mechanisms of aging in the PD immune microenvironment, inspiring more effective therapeutic methods.

## Data availability statement

The original contributions presented in the study are included in the article/**Supplementary Material**. Further inquiries can be directed to the corresponding author.

## Ethics statement

The studies involving human participants were reviewed and approved by Ethics Committee of Stomatological Hospital of Chongqing Medical University (NO:2020 LSNo.79). The patients/participants provided their written informed consent to participate in this study.

## Author contributions

XZ: Conceptualization; design. LP and HC: Formal analysis, visualization, manuscript writing. ZW: Sample collection. YH: Writing – review & editing. All authors contributed to the article and approved the submitted version.

## Funding

This work was financially supported by the National Natural Science Foundation of China (81700982) and the Chongqing Medical Reserve Talent Studio for Young People (ZQNYXGDRCGZS2019004).

## Conflict of interest

The authors declare that the research was conducted in the absence of any commercial or financial relationships that could be construed as a potential conflict of interest.

## Publisher’s note

All claims expressed in this article are solely those of the authors and do not necessarily represent those of their affiliated organizations, or those of the publisher, the editors and the reviewers. Any product that may be evaluated in this article, or claim that may be made by its manufacturer, is not guaranteed or endorsed by the publisher.

## Supplementary material

Supplementary Figure 1 Diversity of immune microenvironment characteristics between periodontitis and healthy samples. **(A)** The proportion of cell infiltration in 22 types of immune cells in periodontitis and healthy samples. **(B)** Box plots showing the differences in immune cell infiltrations in periodontitis and healthy samples. **(C-E)** The activity differences of each immune reaction gene-set, the expression differences of each HLA gene and the expression differences of each immune checkpoint between periodontitis and healthy samples, respectively.

Supplementary Table 1 Characteristics of selected GEO datasets.

Supplementary Table 2 Three hundred seven genes related to aging.

Supplementary Table 3 The detail of clinical information on eight patients.

Supplementary Table 4 The primer sequences for qRT-PCR.

Supplementary Table 5 Differentially Expressed Aging Related Genes.

Supplementary Table 6 The output results of the neural network model.

Supplementary Table 7 Aging modification patterns.

Supplementary Table 8 Aging related genes in periodontitis.

Supplementary Table 9 Module-subtype correlations.
