# Supplementary figures and images for: Identification and validation of a classifier based on hub aging-related genes and aging subtypes correlation with immune microenvironment for periodontitis
Source: Front Immunol. 2022 Nov 1;13:1042484. doi: 10.3389/fimmu.2022.1042484 (PMC9663931; doi:10.3389/fimmu.2022.1042484)

A

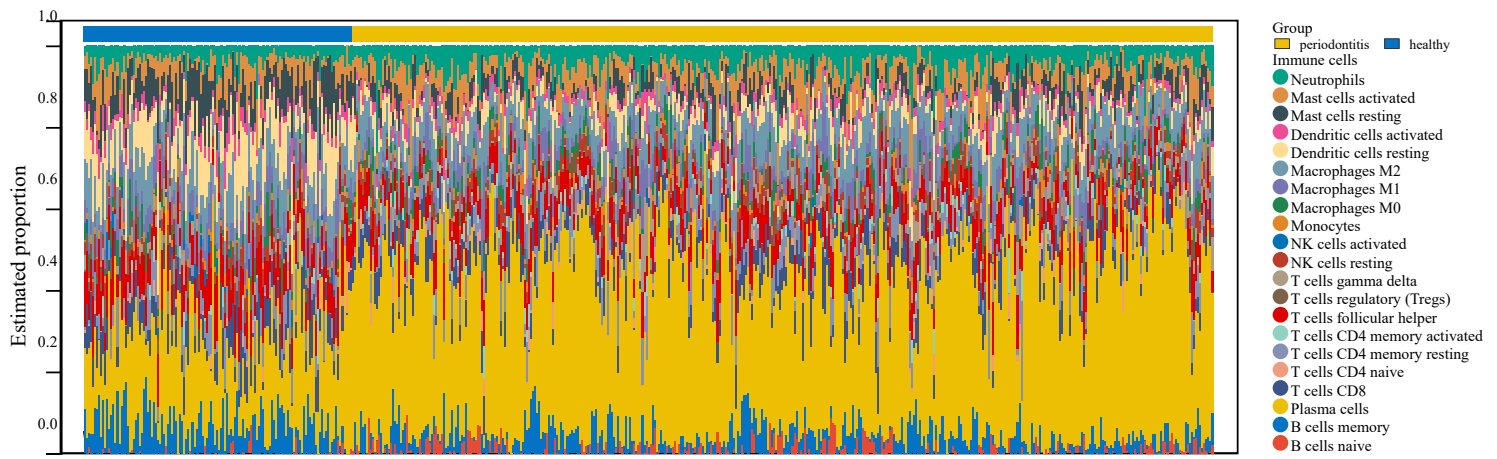

B

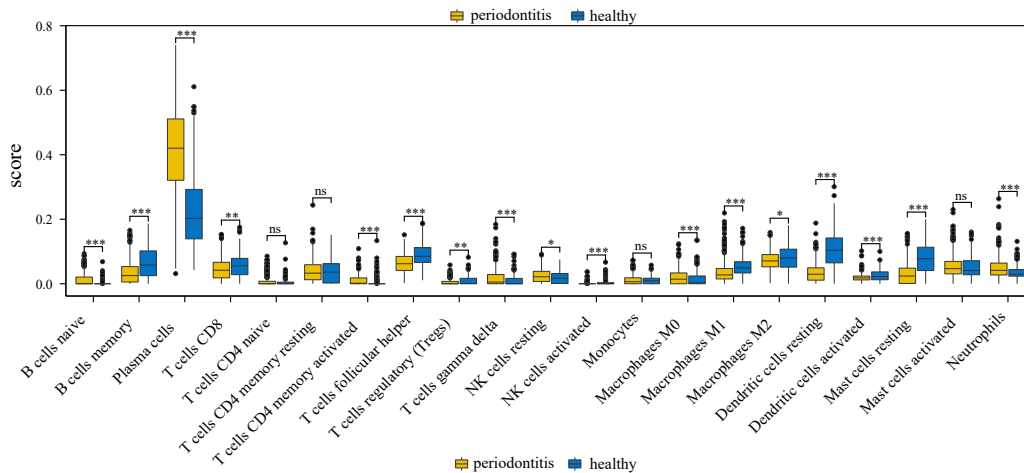

C

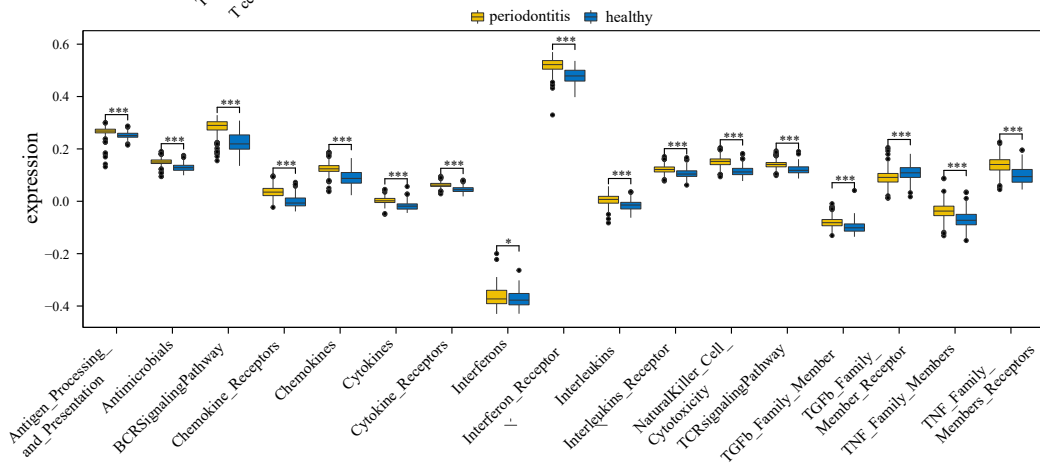

D

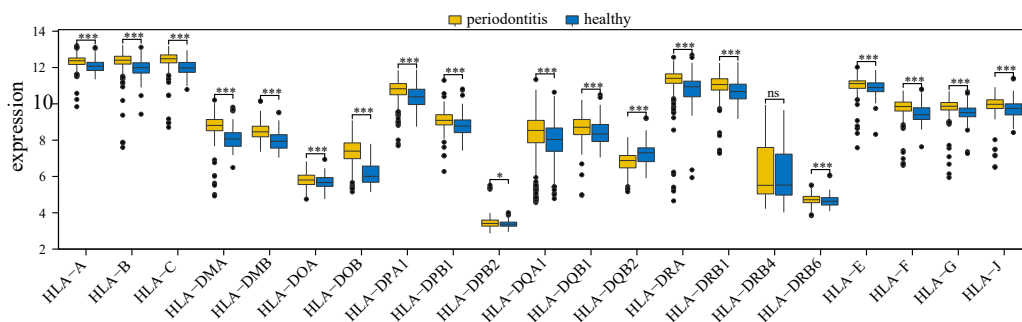

E

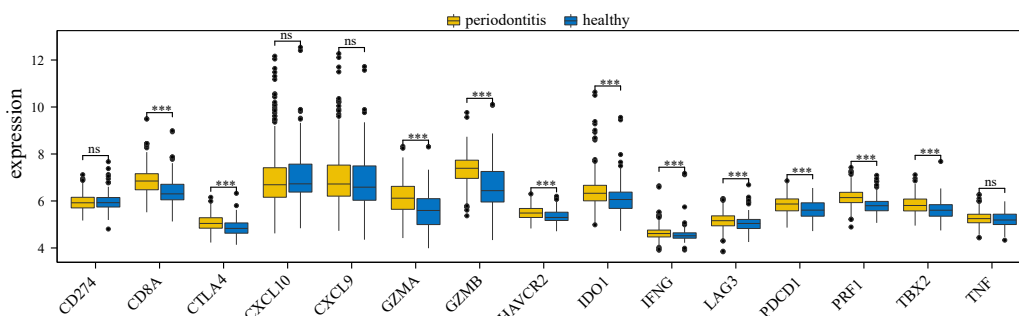

Supplement: Supplementary Figure 1 — Diversity of immune microenvironment characteristics between periodontitis and healthy samples. (A) The proportion of cell infiltration in 22 types of immune cells in periodontitis and healthy samples. (B) Box plots showing the differences in immune cell infiltrations in periodontitis and healthy samples. (C-E) The activity differences of each immune reaction gene-set, the expression differences of each HLA gene and the expression differences of each immune checkpoint between periodontitis and healthy samples, respectively. [file Image_1.pdf]
